# Monitoring the Effect of Calcium Nitrate on the Induction Period of Cement Hydration via Low-Field NMR Relaxometry

**DOI:** 10.3390/molecules28020476

**Published:** 2023-01-04

**Authors:** Mihai M. Rusu, David Faux, Ioan Ardelean

**Affiliations:** 1 Department of Physics and Chemistry, Technical University of Cluj-Napoca, 400114 Cluj-Napoca, Romania; 2 Department of Physics, University of Surrey, Guildford GU2 7XH, UK

**Keywords:** cement hydration, induction period, accelerator, NMR relaxometry, Fast Field Cycling, 3-Tau model

## Abstract

The hydration process of Portland cement is still not completely understood. For instance, it is not clear what produces the induction period, which follows the initial period of fast reaction, and is characterized by a reduced reactivity. To contribute to such understanding, we compare here the hydration process of two cement samples, the simple cement paste and the cement paste containing calcium nitrate as an accelerator. The hydration of these samples is monitored during the induction period using two different low-field nuclear magnetic resonance (NMR) relaxometry techniques. The transverse relaxation measurements of the ^1^H nuclei at 20 MHz resonance frequency show that the capillary pore water is not consumed during the induction period and that this stage is shortened in the presence of calcium nitrate. The longitudinal relaxation measurements, performed at variable Larmor frequency of the ^1^H nuclei, reveal a continuous increase in the surface-to-volume ratio of the capillary pores, even during the induction period, and this increase is faster in the presence of calcium nitrate. The desorption time of water molecules from the surface was also evaluated, and it increases in the presence of calcium nitrate.

## 1. Introduction

There are various applications of cement-based materials where the process of cement hydration requires acceleration. Some examples include urgent repair works, an increase in the productivity of precast concrete elements, or 3D printing technology [1]. An accelerated hydration process can be obtained by raising the curing temperature [2] using a cement powder of higher fineness [3] by introducing calcium silicate hydrate seeds [4] or by adding different types of accelerators [5,6]. One such accelerator is calcium nitrate (Ca(NO_3_)_2_), which is used for its corrosion inhibitor properties as a substitute for calcium chloride in steel-reinforced concrete structures [7].

The hydration of Portland cement is a complicated chemical reaction that starts immediately after mixing the cement grains with water molecules and is characterized by five evolution intervals: initial period, induction period, acceleration period, deceleration period, and continuous slow hydration period [8]. A comprehensive description of cement hydration, containing also the cement chemistry terminology and abbreviations, can be found in Refs. [8,9]. The duration of these evolution intervals is strongly influenced by the composition and the size of the cement grains, the amount of water in the mixture, and by the curing temperature [2,3]. Although it has been investigated for a long time, the hydration process is still not fully understood [10,11]. For instance, it is not clear what produces the induction period, which is characterized by a reduced reactivity [12]. There are two main hypotheses used to explain the low reactivity of cement grains during the induction period, the so called protective membrane theory [13,14] and the dissolution theory [11,15], but none of them are fully accepted by the cement research community.

The protective membrane theory [10] assumes that a layer of monosulfoaluminoferrite hydrates (AFm) [8,16] and metastable calcium silicate hydrate (C-S-H) [13] forms on the surface of cement grains during the initial period (first minutes of hydration) and this layer covers the reacting surface, thus preventing the fast dissolution and the appearance of the induction period. At the end of the induction period, a stable but permeable C-S-H forms from the precipitation of the solution, and the metastable layer disappears; thus, water access to the surface is improved, and the hydration process is accelerated. The existence of such a layer is questioned by some authors, especially because it has not yet been directly visualized despite the progress of microscopic techniques [10].

Dissolution theory assumes that the dissolution rate of a crystalline material is influenced by its interfacial properties and the surrounding solution [15]. The interfacial properties of the crystalline material depend on chemical composition, type of bond, impurities, and lattice defects. The solution properties depend on the nature of the solvent, ionic composition, or temperature and are characterized by the so called undersaturation, which reflects deviation from equilibrium [15]. In the case of the cement clinker, the dissolution rate of alite (C_3_S), the most abundant component of the clinker, decreases as undersaturation of the system decreases by moving toward equilibrium [11,15]. Thus, at very high undersaturation levels, immediately after placing water molecules in contact with C_3_S, vacancy islands nucleate on the surface of C_3_S. As undersaturation decreases it is not possible to create etch pits on plain surfaces but only on defects such as dislocations. At lower levels of undersaturation, etch pits can be created only at a lower rate, and the slow dissolution occurs, thus the system is in the induction period. According to this model, the onset of the acceleration period occurs when a high enough concentration of Ca^2+^ ions is present in solution, and portlandite (CH) can precipitate. Portlandite precipitation increases the degree of undersaturation and thus determines an accelerated C-S-H precipitation [15].

The presence of an accelerator complicates the hydration process but can also help us understand it better [6]. If the accelerator is calcium nitrate, its presence affects both the hydration of tricalcium aluminate (C_3_A) and tricalcium silicate (C_3_S). Thus, the presence of calcium nitrate may lead to an increased formation of mono-sulfoaluminoferrite hydrates (AFm) and trisulfoaluminoferrite hydrates (AFt), in which the most important phase is ettringite [8,17], and accelerates C_3_S reaction due to the fast dissolution of Ca(NO_3_)_2_, which produces a faster supersaturation with Ca^2+^ ions of the solution, which in turn produces earlier crystallization of CH [11,15].

The influence of accelerators, or other admixtures, on the hydration process can be studied by a combination of different techniques, such as calorimetric studies [18], X-ray diffraction [19], mercury porosimetry [20], scanning electron microscopy [21], or ultrasonic tests [22]. Some of these techniques require special sample preparation or stopping of the hydration process when utilized. That is why, when studying water evolution and pore structure development inside cement-based materials, it is favorable to use ^1^H low-field nuclear magnetic resonance (NMR) relaxometry techniques [3,22,23,24,25,26]. The advantage of NMR relaxometry over other techniques is that it allows nonperturbative investigations even during the hydration process without the requirement to stop the hydration process and without any pretreatment of the sample.

The most applied variant of NMR relaxometry is based on transverse relaxation measurements using the well-known Carr-Purcell-Meiboom-Gill (CPMG) [27] pulse sequence. This technique provides the relaxation time distribution of water molecules and, thus, information about molecule–surface interaction, saturation degree, and the pore size distribution. Another technique that can be successfully applied to cement-based materials is the Fast Field Cycling (FFC) relaxometry [2,28,29,30]. The technique allows the determination of longitudinal relaxation time as a function of the proton Larmor frequency (10 kH–10 MHz in the present work), thus becomes sensitive to a wider range of molecular mobilities compared to the measurements performed at a single frequency [28].

In the present work, both CPMG and FFC NMR relaxometry will be applied to study the influence introduced by an accelerator on the hydration dynamics of cement paste during the induction period. In the case of the FFC NMR relaxometry technique, the relaxation dispersion curves recorded at different hydration times for the simple cement paste and the cement paste containing the accelerator will be evaluated using the 3-Tau relaxation model [31,32,33,34,35]. By comparing the outcomes of the two techniques, information about the interaction of water molecules with the surface of cement grains and about surface evolution during the induction period will be extracted.

## 2. Results and Discussions

To monitor water evolution during cement hydration, CPMG experiments were performed first (see Section 3). The two cement-based materials under investigation were the simple cement paste (CP) and the cement paste containing the accelerator (CP+2% Ca(NO_3_)_2_). The relaxation decay curves were formed of 1000 echoes recorded for short echo time intervals of 0.08 ms, both for reducing the internal gradient effects and detecting short relaxation time components present in the sample. The recorded CPMG echo trains were then used to extract the relaxation time distributions using a numerical Laplace transformation [36,37].

**Figure 1:**
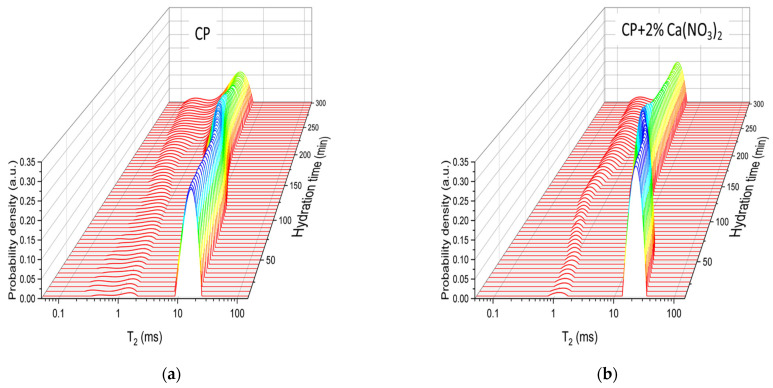
Relaxation time distributions of the two cement pastes during the early hydration: (**a**) the simple cement paste CP; (**b**) the cement paste prepared with 2% accelerator (CP + 2% Ca(NO_3_)_2_), by cement weight. The smaller peak corresponds to the embedded water while the larger peak corresponds to the capillary water.

Figure 1 shows the relaxation time distributions of the simple cement paste (Figure 1a) and the cement paste containing the accelerator (Figure 1b). The first CPMG data were recorded at 10 min from mixing start and the last at 300 min. One can observe a distribution of relaxation times with two peaks corresponding to two distinct water environments in the mixture. The first peak, arising at about 1 ms, can be associated to the embedded water inside the flocculation of the cement grains. A second peak of higher area and longer $T_{2}$ (between 10–30 ms) corresponds to the capillary pore water. As can be noticed, both peaks evolve during hydration with a faster evolution in the case of the sample containing 2% of Ca(NO_3_)_2_, by cement mass. The decrease in the area of the capillary water peak during hydration can be associated with the transformation of the capillary water into solid components such as C-S-H, ettringite, and CH (not visible in a low-field NMR experiment due to the short relaxation times). Note that, the porous structure of C-S-H also contains water molecules of lower mobility, the so-called gel water which manifests itself by an increase in the area of the first peak after a certain hydration time. However, here we will concentrate only on the capillary water peak because it is also studied in the FFC NMR relaxation measurements.

**Figure 2:**
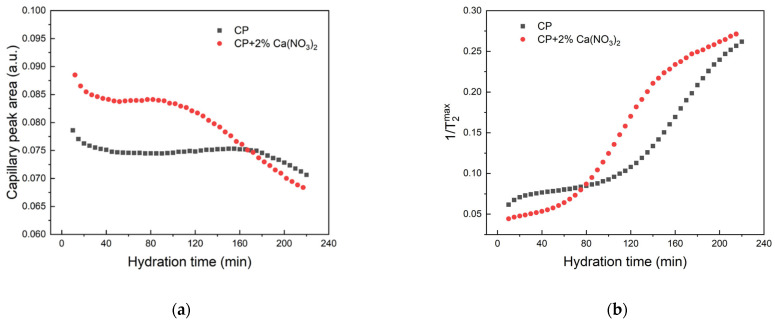
(**a**) Capillary peak area versus hydration time for the two cement pastes. (**b**) Relaxation rates corresponding to the maximum of the capillary versus hydration time.

To quantify the consumption of capillary water and the pore size evolution during hydration, we have evaluated in Figure 2 the capillary peak area (Figure 2a) and the relaxation rate $1/T_{2}^{\max}$ corresponding to the position of the peak maximum (Figure 2b). We restricted our evaluation to hydration times up to 240 min, when one can clearly separate the capillary water component from the gel water (intra and inter C-S-H) [38]. As can be observed in Figure 2a, the peak area decreases in the first 20 min (initial period) for both samples, indicating a fast water consumption and the rapid transformation into hydration products [6,10]. However, after 40 min of hydration the reaction seems to stop, and no capillary water consumption is observed after this time until 160 min in the case of simple cement paste (CP) and 80 min in the case of cement paste containing the accelerator (CP + 2% Ca(NO_3_)_2_). This behavior demonstrates the effect of shortening the induction period of cement hydration in the presence of calcium nitrate. It also demonstrated an acceleration of the hydration kinetics in the presence of calcium nitrate.

If we compare the relaxation times corresponding to the capillary peak maximum (Figure 2b), we notice a continuous variation of the relaxation time for both samples, and this variation is again accelerated in the presence of Ca(NO_3_)_2_. However, if we compare the relaxation rates (Figure 2b) with the capillary water evolution (Figure 2a), we notice that a variation of the relaxation time may exist without water consumption. According to Equation (2) (see Section 3), there are two reasons for such an evolution: increase in the surface-to-volume ratio of the pores and increase in surface relaxivity. However, separation of these contributions is not possible in a CPMG experiment. That is why we complete here the investigations on the hydration of the two samples with Fast Field Cycling NMR relaxation measurements, which better separate the two contributions, provided that a suitable relaxation model is used [31,32].

Longitudinal relaxation measurements were performed on both samples using the FFC techniques described in Section 3. The switching time of 2 ms was set to reduce the contribution of the embedded water component to the detected signal and to ensure that only the capillary water is detected. Due to the required time for the adjustment of the instrument parameters, the first relaxation dispersion curve could be detected at 20 min from mixing start. The subsequent measurements were performed every 30 min, up to 170 min. The last measurement was recorded at 170 min due to the fact that, after this hydration time, it is difficult to separate the embedded water from capillary water contribution, as one can directly observe in Figure 1.

**Figure 3:**
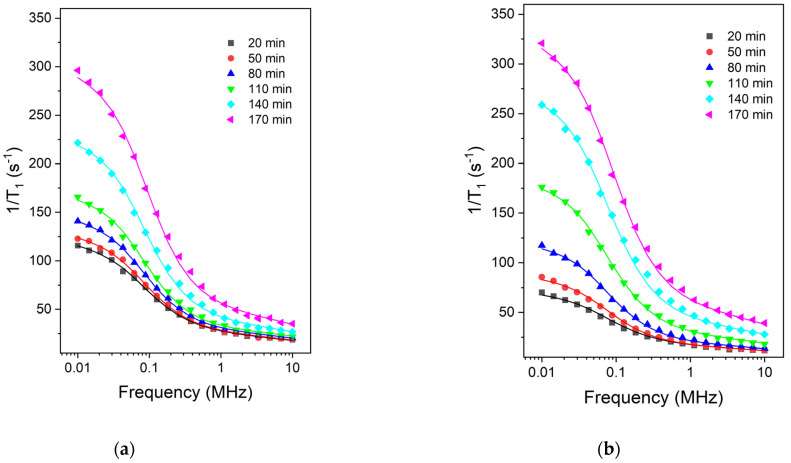
Relaxation dispersion curves recorded for the two samples during the early hydration: (**a**) the simple cement paste CP; (**b**) the cement paste prepared with 2% accelerator (CP + 2% Ca(NO_3_)_2_). The hydration times are indicated in the legend. The lines represent the best fits obtained with the 3-Tau model.

The relaxation dispersion curves recorded for the two samples at different hydration times are shown in Figure 3. One can observe a faster evolution in the case of the sample containing 2% of Ca(NO_3_)_2_, by cement mass (Figure 3b) as compared to the simple cement paste (Figure 3a) and this evolution takes place at all frequencies. To extract information on the dynamics of confined molecules, the relaxation dispersion curves were fitted with the 3-Tau model [31,32,33,34,35], briefly described in Section 3.3. The fitting approach follows the comprehensive description in Ref. [39] and uses the software package provided by Kogon and Faux [40]. The continuous lines represent the best fits of the experimental data with the 3-Tau model. On this basis the following fitting parameters could be extracted (see Section 3.3): $\tau_{l}$-surface diffusion time; $\tau_{b}$-bulk diffusion time; $\tau_{d}$-desorption time; $N_{par}$-number density of paramagnetic ions; Sδ/V-surface layer volume to pore volume ratio.

To fit the relaxation dispersion curves with the 3-Tau model, we started the fitting approach by setting all five parameters free. However, after several fitting rounds, we noticed that two of them, $\tau_{l}$ and $\tau_{d}$, do not vary and can be kept constant during the first 170 min of hydration. Thus, the surface diffusion time of $\tau_{l}=0.24\;\mu s$ and the bulk diffusion time of $\tau_{b}=17.8\;\mathrm{ps}$ could be considered fixed for both samples and all hydration times. The desorption time parameter $\tau_{d}$ is, however, different in the case of CP sample as compared with the CP + 2% Ca(NO_3_)_2_ sample. Thus, for hydration times of up to 140 min the desorption time value $\tau_{d}=3.65\;\mu s$ was extracted in the case of cement paste and $\tau_{d}=4.22\;\mu s$ for the sample containing the accelerator. The longer desorption time in the case of the sample containing the accelerator shows a stronger interaction of water molecules with the surface. Note, however, that, for the hydration time of 170 min, both samples revealed the same value of desorption time $\tau_{d}=3.16\;\mu s$. This indicates identical surface properties of the two samples at 170 min of hydration. The surface diffusion time $\tau_{l}$ for the two samples during the interval 20–140 min allows the extraction of a surface diffusion coefficient $D_{l}=5.06\times 10^{-14}\;m^{2}/s$ that is three orders of magnitude smaller than that extracted on the basis of Korb’s relaxation model [29]. The surface water diffusion coefficient found here is typical of “hard” solid porous material such as pastes (plaster/silica), clay, and rocks independent of the model [33]. The diffusion coefficient is low because the surface layer of water is both chemi- and physiosorbed to the surface. The surface water can diffuse by breaking away from the surface before moving over the potential energy barrier to a neighboring vacant site. However, most surface sites are filled unless a water molecule has desorbed into the bulk to create a vacancy. Desorption itself is hindered by the blocking effect of the mobile water in the second hydration layer.

**Figure 4:**
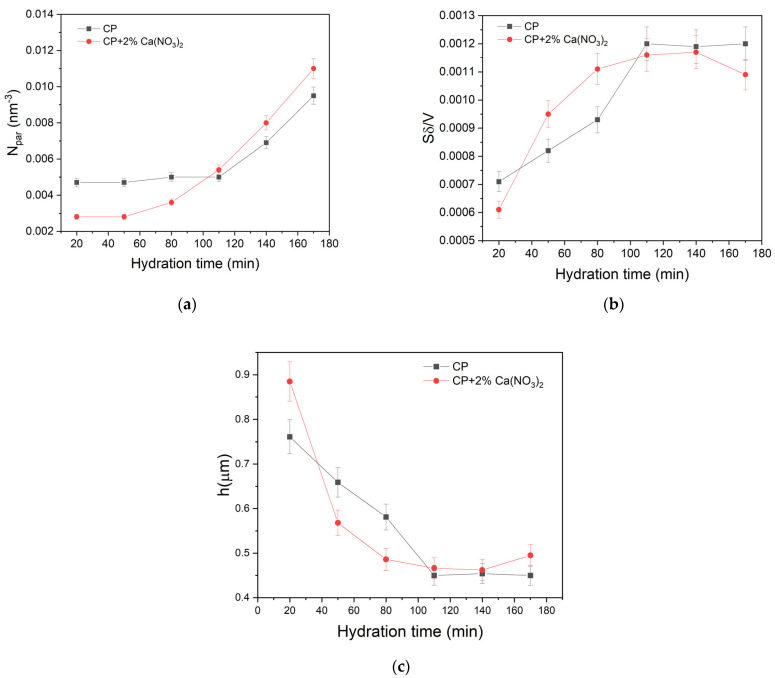
The fitting parameters, extracted from the data in Figure 3 by using the 3-Tau model. (**a**) Number density of paramagnetic ions versus hydration time. (**b**) The ratio the ratio between the volume of the surface layer and the pore volume versus hydration time. (**c**) The pore size versus hydration time in the approximation of plane pores.

The number density of paramagnetic ions $N_{par}$ of the two samples was also determined from the fitting approach. The values are represented in Figure 4a as a function of hydration time. One can observe a dependence of this parameter on hydration time for both samples. However, even if the cement pastes are made of identical cement powders, the smaller number densities are observed for the sample containing the accelerator at initial hydration times. That behavior could be explained by the presence of a thicker layer of hydration products, which isolates the capillary water from the surface [6], which is equivalent with a smaller effective ion density. Another observation is that a rapid increase in $N_{par}$ occurs at hydration times of about 100 min (CP) and 50 min, respectively (CP + 2% Ca(NO_3_)_2_). This increase in $N_{par}$ can be associated with an increase in the permeability for water molecules to reach the grain surface and the beginning of the acceleration process. We notice that the hydration times for which the increase in $N_{par}$ is observed in Figure 4a corresponds to the hydration times when changes in the relaxation rate behavior (Figure 2b) are observed, that is the end of induction stage.

Another parameter that can be extracted from the fitting approach is Sδ/V, which represents the ratio between the volume of the surface layer and the pore volume. Figure 4b shows an increase of the surface volume to pore volume ratio during the induction period. The increase of Sδ/V during induction period can be associated with a continuous increase of the pore surface due to continuous formation of etch pits on the surface of cement grains [15]. The formation of these pits does not consume the capillary water as can be observed from the constant capillary peak area (Figure 2a). A similar conclusion about the Sδ/V increase was drawn from a fractal analysis of the pore surface based on transverse NMR relaxation measurements [24]. Figure 4b shows a faster saturation of the increase in the Sδ/V in the presence of the accelerator (CP + 2% Ca(NO_3_)_2_, by cement mass), as compared with the simple cement paste (CP). The dependence of Sδ/V parameter on hydration time allows, in the approximation of plane pores, extraction of an effective pore size h. The dependence of the pore size on hydration time is shown in Figure 4c. Note, however, that the extracted values are affected by the roughness of the pore surface and must be considered with caution. Nevertheless, the extracted values are in the same range of dimensions as those obtained on similar samples using a different approach, based on diffusion in internal gradients [41].

## 3. Materials and Methods

### 3.1. Sample Preparation

Two cement-based samples were prepared using white Portland cement (CEM I 52.5R Holcim, Romania), for a water-to-cement ratio of 0.4, by weight. The chemical composition of the cement powder was determined in Ref. [23] and is specific to a white Portland cement with low iron content: CaO (70.7%), SiO_2_ (16.3%), and Al_2_O_3_ (4.7%), with gypsum derived sulphate (5.8%) and other impurities based on MgO (0.9%), Na_2_O (0.7%), KO (0.6%), and Fe_2_O_3_ (0.3%). The grain size distribution indicates an average size and a maximum size of 5 μm and 25 μm, respectively. The white cement, with a low content of iron oxide, was chosen here with the aim of reducing the internal gradient effects on echo attenuation in the CPMG experiment [42]. The first sample was a simple cement paste (CP) obtained by mixing the cement grains with distilled water. The second sample further contains the accelerator (Ca(NO_3_)_2_, acquired from Nordic Chemicals SRL, Romania). The amount of accelerator is 2%, by cement mass, and was introduced into the mixture by first dissolving it in water. The ingredients were mixed at room temperature for 5 min using a mechanical mixer. The first NMR experiments started at 10 min from the beginning of mixing in the case of CPMG measurements and at 20 min in the case of FFC relaxometry measurements. They were performed under identical temperature conditions at 35 °C.

### 3.2. The NMR Methodology

NMR relaxation measurements of the molecules confined inside porous media provide information about the porous structure, the molecule-surface interaction, and the liquid distribution inside pores. To extract such information, two types of experiments are usually performed: i) transverse relaxation time distribution determinations for confined molecules and ii) longitudinal relaxation measurements of ^1^H nuclei as a function of Larmor frequency. Of course, these experiments must be supplemented with theoretical models describing relaxation phenomena under specific experimental conditions. Here, we will shortly describe the two experiments performed in our investigations and the specific relaxation model.

**Figure 5:**
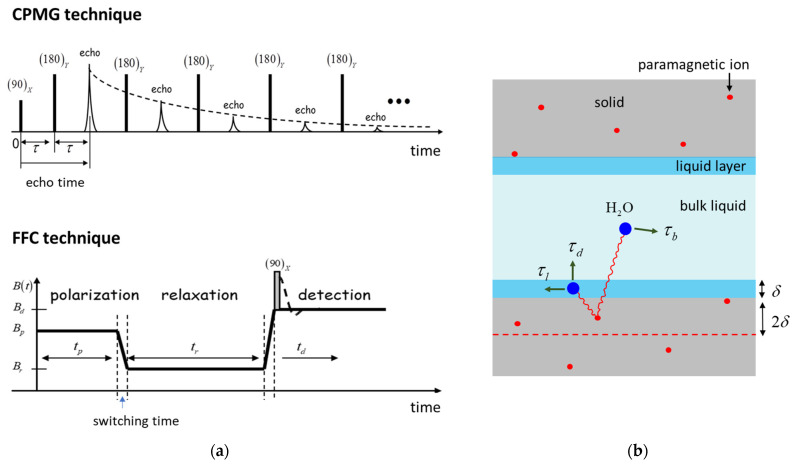
**(a)** Top**:** CPMG pulse sequence generating the echo trains for transverse relaxation measurements. Bottom: The variation of the main magnetic field along a Fast Field Cycling relaxation experiment. (**b**) The schematic representation of the interactions accounted in the frame of the 3-Tau model [32].

Transverse relaxation time distributions can be obtained from the echo trains recorded using the well-known CPMG pulse sequence shown in Figure 5a (top). The pulse sequence consists of a series of hard 180-degree radiofrequency (RF) pulses following a first 90-degree pulse. An echo train is composed of the echoes recorded at evolution intervals t=2nτ. The amplitude of the *n*-th echo in the echo train attenuates according to the formula [38]:

(1) $$A_{n}=A_{0}\int_{0}^{\infty }P\left(T_{2}\right)e^{-\frac{2n\tau }{T_{2}}}dT_{2}$$

where $A_{0}$ is a constant affected by the sample magnetization, temperature, and hardware characteristics of the NMR instrument. $P\left(T_{2}\right)$ represents the relaxation time probability density, and $T_{2}$ is the relaxation time of molecules confined inside a given pore. As the above formula suggests, a numerical Laplace inversion of the recorded echo train amplitudes provides the relaxation time distribution [36,37].

The transverse relaxation time depends on the surface-to-volume ratio S/V of the pore by the formula [38]

(2) $$\frac{1}{T_{2}}=\frac{1}{T_{2}^{bulk}}+\rho \frac{S}{V}$$

where $1/T_{2}^{bulk}$ represents the relaxation rate of the bulk-like liquid inside the pores, ρ represents the relaxivity, a constant depending on the paramagnetic impurity content of the pore surface; wettability of the filling liquid; temperature; and the strength of the main magnetic field of the instrument. Considering the above equation, the relaxation time distribution extracted via Equation (1) allows the determination of the pore size distribution, provided that the relaxivity is known from an independent experiment. Even if the relaxivity is not known, the relative distribution of pore sizes can be obtained. Note that, the above equation applies only for saturated pores and by neglecting diffusion effects on echo train attenuation [38,42].

Frequency dependent longitudinal relaxation measurements can be performed with the so-called Fast Field Cycling technique [28]. This technique allows the polarization and detection of the nuclear spins at higher fields while relaxation can take place at lower fields. With this approach, a significant increase in the detection sensitivity can be obtained as compared with the case in which polarization and detection would be performed at lower fields that are identical to the relaxation field. Thus, a main advantage of the FFC technique is the possibility of making longitudinal relaxation measurements at different Larmor frequencies (10 kHz–10 MHz, in the present work).

The schematic representation of an FFC experiment is shown in Figure 5a (bottom) and consists of three evolution intervals: polarization, relaxation, and detection [28]. Thus, during the polarization interval, the sample polarizes in a higher field $B_{p}$ for a duration $t_{p}$ until it reaches saturation. Then the magnetic field is rapidly switched to a value $B_{r}$ and the nuclear spins relax inside this field for a relaxation time $t_{r}$. Immediately after, the field is switched up to a detection field $B_{d}$ and the remaining magnetization is measured by applying a 90-degree RF pulse. Note that the switching time from one field to another cannot be made arbitrarily short and that limits the detection of very short relaxation components. On the other side, the switching time can be used as a filter for short relaxation times, as was done in our experiments.

The CPMG relaxation measurements were performed using a low-field NMR instrument (Minispec MQ20, Bruker BioSpin GmbH, Rheinstetten, Germany), operating at a resonance frequency of 20 MHz. This low frequency value, together with the short echo time interval, provides the conditions for reduced diffusion effects on echo train attenuation and thus justifies the implementation of Equation (2) for the relaxation rate [38,42]. A commercially available Fast Field Cycling NMR relaxometer (SMARtracer, Stelar S.R.L, Mede, Italy) was used for recording the relaxation dispersion curves in a range of proton resonance frequencies between 10 kHz and 10 MHz. All the measurements were performed at 35 °C.

### 3.3. The Relaxation Model

In the case of CPMG relaxation measurements, the frequency dependence of the transverse relaxation rate is not important. This is because the measurements are performed at a single proton resonance frequency (here, 20 MHz) and a simple relation, Equation (2), connects the surface-to-volume ratio with the relaxation time. However, in the case of the FFC relaxation measurements, which provide the dependence of the longitudinal relaxation rate on the Larmor frequency (the so-called relaxation dispersion curves), a relaxation model, adapted to the system studied, must be used to extract the dynamical information. A valuable model describing relaxation of water molecules confined inside the pores of cement-based materials is the 3-Tau model developed by Faux and collaborators [31,32,33,34,35]. This model was comprehensively discussed in the previous publications of Faux and collaborators [31,32,33,34,35] and here we will only summarize its main features and outcomes. Moreover, to facilitate the applications of the 3-Tau model to real samples a fitting package was provided and tested on different samples by Faux and collaborators [39,40].

In the frame of the 3-Tau model, the relaxation rate of water molecules confined inside porous materials containing paramagnetic impurities is dominated by the thermally modulated dipolar interactions of the proton spins with the paramagnetic ions (here Fe^3+^) inside the solid matrix. According to the 3-Tau model shown in Figure 5b, water molecules can be found in two distinct environments: in the bulk-like state and in a surface monolayer of thickness δ=0.27 nm (the size of one water molecule). These molecules encounter displacements along the surface and in bulk (indicated by arrows on Figure 5b), which modulate the dipolar interaction between the nuclear spins and the electronic spin of the paramagnetic ions inside the solid structure. As a result of these modulations the longitudinal relaxation rate is a complicated function of three-time constants (3-Tau), the density of paramagnetic ions and the surface-to-volume ratio:

(3) $$\frac{1}{T_{1}\left(\omega \right)}=f\left(\tau_{l},\tau_{b},\tau_{d},N_{para},\frac{S\delta }{V}\right)$$

In the above expression, $\tau_{l}$ is a characteristic time that describes the displacement of water molecules on the surface and is related to the surface water diffusion coefficient $D_{l}$ by the formula $\tau_{l}=\delta^{2}/6D_{l}$. The bulk-like water molecules are characterized by a diffusion time constant $\tau_{b}$ which describes the diffusional displacement of bulk molecules over a distance δ. It is related to the bulk diffusion coefficient $D_{b}$ by the formula $\tau_{b}=\delta^{2}/6D_{b}$. Note that, in the case of pure water, at room temperature, $\tau_{b}=5.3\;\mathrm{ps}$ but it would be longer for water confined inside the pores of cement paste due to the presence of dissolved ions, which interact with water and hinder diffusion, but also due to their slower mobility inside the second hydration layer at the surface, which dominates the bulk relaxation [39]. Assuming that the surface spins desorb as $\exp\left(-t/\tau_{d}\right)$, the third constant $\tau_{d}$ characterizes the time water molecule spend at the surface before desorption. According with molecular dynamic simulations $\tau_{d}$ and $\tau_{l}$ should be in the same order of magnitude [31].

The number of paramagnetic ions per unit volume $N_{para}$ is considered here as an effective parameter corresponding to the position of an effective layer (dashed line in Figure 5b located 2δ under the surface of the pores. Note that if a layer of hydration products precipitates on the surface of cement grains, $N_{para}$ should only indirectly depend on the paramagnetic impurity content of the cement grains. Sδ/V represents the ratio between the volume of the surface layer and the pore volume. Assuming planar pores, this ratio allows finding of another key quantity, the pore size h as the distance between the two planes. However, in the case of an evolving porous structure, as is the case of cement-based materials, the change in the surface to volume ratio can be also determined by the change in fractal dimension [24], and the pore size parameter extracted from the fitting approach must be used here with caution when characterizing the pore size evolution during hydration.

## 4. Conclusions

Two low-field NMR relaxometry techniques were implemented to extract information about the influence of calcium nitrate on early hydration of a white cement paste. Transverse relaxation measurements, performed with CPMG technique, have demonstrated that water contained inside the capillary pores is not consumed during the induction period. However, the duration of the induction stage is shortened in the presence of calcium nitrate. Longitudinal relaxation dispersion curves, recorded with the FFC NMR relaxometry technique, could be well-fitted with the 3-Tau relaxation model for all hydration times. The results of the fitting revealed a continuous increase in the surface-to-volume ratio of the capillary pores, even during the induction period, and this increase is faster in the presence of calcium nitrate. It was also observed that the desorption time of water molecules from the capillary pore surface increases in the presence of calcium nitrate but is constant during the induction period.

## Data Availability

Data may be provided on request from corresponding author.
